# Soft Sub‐Structured Multi‐Material Biosensor Hydrogels with Enzymes Retained by Plant Viral Scaffolds

**DOI:** 10.1002/mabi.202300311

**Published:** 2023-11-15

**Authors:** Jana Grübel, Tim Wendlandt, Daniela Urban, Corinna O. Jauch, Christina Wege, Günter E. M. Tovar, Alexander Southan

**Affiliations:** ^1^ Institute of Interfacial Process Engineering and Plasma Technology IGVP University of Stuttgart Nobelstr. 12 70569 Stuttgart Germany; ^2^ Institute of Biomaterials and Biomolecular Systems University of Stuttgart Pfaffenwaldring 57 70569 Stuttgart Germany; ^3^ Fraunhofer Institute for Interfacial Engineering and Biotechnology IGB Nobelstr. 12 70569 Stuttgart Germany; ^4^ Max Planck Institute for Intelligent Systems Heisenbergstr. 3 70569 Stuttgart Germany

**Keywords:** bioconjugation, biosensing, enzymes, hydrogels, inkjet printing, tobacco mosaic virus

## Abstract

An all‐soft multi‐material combination consisting of a hydrogel based on poly(ethylene glycol) (PEG) coated with spatially defined spots of gelatin methacryloyl (GM) containing selectively addressable viral nanorods is presented, and its basic application as a qualitative biosensor with reporter enzymes displayed on the tobacco mosaic virus (TMV) bioscaffolds within the GM is demonstrated. Biologically inert PEG supports are equipped with GM spots serving as biological matrix for enzymes clustered on TMV particles preventing diffusion out of the gel. For this multi‐material combination, i) the PEG‐based hydrogel surface is modified to achieve a clear boundary between coated and non‐coated regions by introducing either isothiouronium or thiol groups. ii) Cross‐linking of the GM spots is studied to achieve anchoring to the hydrogel surface. iii) The enzymes horseradish peroxidase or penicillinase (Pen) are conjugated to TMV and integrated into the GM matrix. In contrast to free enzymes, enzyme‐decorated TMVs persist in GM spots and show sustained enzyme activity as evidenced by specific color reaction after 7 days of washing, and for Pen after 22 months after dry storage. Therefore, the integration of enzyme‐coupled TMV into hydrogel matrices is a promising and versatile approach to obtaining reusable and analyte‐specific sensor components.

## Graphical abstract

An all‐soft biosensor consisting of a poly(ethylene glycol)‐based hydrogel coated with spots of gelatin methacryloyl (GM) is generated. The enzymes horseradish peroxidase and penicillinase are conjugated to tobacco mosaic virus (TMV) nanoparticles and integrated into the GM spots. Enzyme‐coupled TMV remains captured in GM spots with sustained sensing activity, in contrast to enzymes incorporated without TMV carriers that diffuse out.

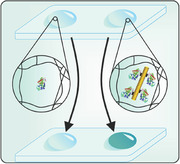

## 1 Introduction

Hydrogels, typically used in the fields of tissue engineering and drug delivery,^[^ ^1^, ^2^, ^3^, ^4^ ^]^ are also suitable as components in bioprinting, soft robotics, or biosensors due to their advantageous properties like biocompatibility, transparency, and ion conductivity.^[^ ^5^, ^6^, ^7^, ^8^ ^]^ Two common polymers to prepare hydrophilic polymer networks are poly(ethylene glycol) (PEG) and gelatin methacryloyl (GM). PEG is well known for its non‐adhesive and protein‐repellent properties,^[^ ^9^, ^10^, ^11^, ^12^ ^]^ and is widely used, for example, as a shell in core–shell structures for long‐circulating drug delivery systems and in the large‐scale precipitation of proteins, for example, for antibody preparations.^[^ ^13^, ^14^, ^15^, ^16^, ^17^, ^18^, ^19^, ^20^ ^]^ Apart from this, PEG is also applied for various biosensor applications.^[^ ^21^, ^22^, ^23^, ^24^ ^]^ GM is often used in the fields of tissue engineering and bioprinting as it is derived from collagen, the main component of the extracellular matrix,^[^ ^25^, ^26^, ^27^ ^]^ and the properties of cross‐linked GM hydrogels also make them an appropriate material for soft sensor applications. However, cross‐linking of thin GM hydrogels in contact with the atmosphere is challenging due to oxygen inhibition and fast drying.^[^ ^28^ ^]^ GM and PEG can be combined, which is typically used to tailor the mechanical properties of hydrogels by varying the ratio of PEG and GM.^[^ ^29^, ^30^, ^31^ ^]^ In a more advanced example from the field of soft robotics, Shin et al. demonstrated that a combination of micropatterned PEG diacrylate (PEGDA) and GM hydrogels with embedded carbon nanotubes and a gold microelectrode can be used for a design inspired by a stingray.^[^ ^32^ ^]^

For sensing applications, hydrogels can interact with enzymes. For example, Ahmad et al. used peptide‐cross‐linked PEG as a biosensor compound to detect collagenase, which plays an important role in diseases like arthritis.^[^ ^21^ ^]^ They measured the degradation of a PEG film by the enzyme with a quartz crystal microbalance. In another study, microstructures of PEGDA with a high aspect ratio were prepared on glass substrates via photolithography.^[^ ^22^ ^]^ The hydrogels contained either ß‐galactosidase (ß‐Gal) or glucose‐oxidase (GOx) combined with horseradish peroxidase (HRP) to detect analytes by fluorescence. The authors were able to detect multiple substrates simultaneously after preparing microarrays in a two‐step process, in which a first microstructure containing GOx/HRP was generated and afterward a microstructure including ß‐Gal by applying a photomask. Elsewhere, the preparation of microneedles based on PEGDA generated by photolithography was reported.^[^ ^24^ ^]^ GOx and lactose oxidase (LOx) were incorporated in the pre‐polymeric solution which was cross‐linked with UV light. Glucose and lactic acid were detected electrochemically by collecting electrons generated by an enzyme reaction. These are all promising examples of using PEG for enzyme‐driven sensor applications, but they suffer from certain limitations such as low reusability or leaching. In the case of a degradation of the sensor by the analyte enzyme, its reusability is restricted. Additionally, enzyme encapsulation directly into the PEG hydrogel matrix is only possible for very small mesh sizes in order to prevent the leaching of the enzymes. Hence, the immobilization of enzymes in hydrogels can be challenging due to the risk of enzyme loss and, in addition, a decrease in their activity.^[^ ^33^ ^]^

An alternative to the physical entrapment of enzymes is their direct coupling to the hydrogel polymer network by covalent or strong non‐covalent bonds like the biotin‐streptavidin interaction. In this context, stable enzyme encapsulation in GM‐based hydrogels was demonstrated by Dehli and co‐workers by modifying GM via biotinylation followed by specific binding of streptavidin‐enzyme conjugates.^[^ ^34^ ^]^ In this way, streptavidin‐conjugated HRP was bound to GM‐based foams. Makhsin et al. prepared a metal‐clad leaky waveguide as an optical biosensor with a low refraction index waveguide layer.^[^ ^23^ ^]^ For the latter, they used a PEG‐based hydrogel containing *N*‐hydroxy succinimide (NHS) groups immobilized on a silanized titanium oxide surface. The amine‐reactivity of the NHS moieties was used to bind and detect analytes such as glycerol and bovine serum albumin. Although the direct coupling of enzymes to the hydrogel matrix can prevent enzyme leaching, it can be challenging and laborious to introduce the necessary functional groups into the hydrogels.

An attractive and versatile alternative approach would be a combination of physical entrapment and chemical coupling. This can be realized by conjugation of the enzymes on particles that are large enough to be encaged in virtually all hydrogels. An auspicious candidate is the nanorod‐shaped tobacco mosaic virus (TMV). TMV has a length of 300 nm and an outer diameter of 18 nm with 2130 identical coat proteins (CP) assembled helically on a ribonucleic acid genome.^[^ ^35^, ^36^, ^37^ ^]^ Cysteine‐modified TMV (TMV_Cys_) provides a protein surface with a reactive thiol group displayed on every CP subunit.^[^ ^38^ ^]^ TMV_Cys_ was used in various applications^[^ ^39^, ^40^, ^41^, ^42^, ^43^ ^]^ including biosensing where it served as an advantageous carrier for enzymes.^[^ ^39^, ^40^, ^41^ ^]^ It was demonstrated that through the binding of the enzymes to the plant virus particles, an increased enzyme loading and prolonged reusability of the sensors was achieved, compared to conventional immobilization routes.^[^ ^39^, ^40^ ^]^ TMV has also been applied in hydrogels in several laboratories before, mainly in the context of 3D tissue culture in combination with cell adhesion studies with the use of peptides,^[^ ^44^ ^]^ as it is richly available from plant resources and non‐pathogenic or toxic to mammals. Southan et al. have incorporated wildtype TMV and TMV_Cys_ in PEGDA hydrogels before and demonstrated that TMV_Cys_ can be covalently coupled into the hydrogel via the binding of thiols to acrylates.^[^ ^42^ ^]^

**Scheme 1:**
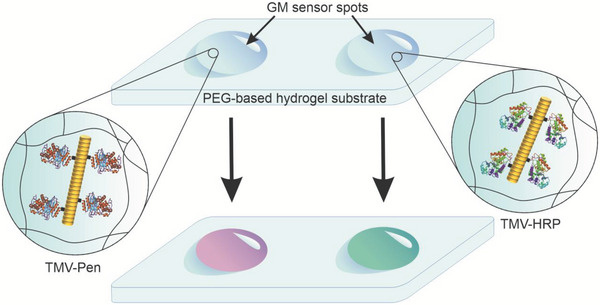
Schematic drawing of the all‐soft multi‐material combination in this study and its basic application as a qualitative biosensor. A substrate based on a humid PEG hydrogel is partially coated with surface‐anchored spots of cross‐linked GM hydrogels. The GM hydrogel spots contain either the enzyme penicillinase (left, TMV‐Pen) or the enzyme horseradish peroxidase (right, TMV‐HRP). Both enzymes are coupled to TMV particles and thus are physically entrapped in the GM hydrogel spots. When exposed to the corresponding substrates of the enzymes, the spots change color (bottom).

In this contribution we therefore aimed to construct a multi‐material setup consisting only of the appropriately functionalized hydrogel materials PEG and GM, supplemented with immobilized TMV as enzyme carrier (**Scheme** **1**), thus decoupling the choice of the hydrogel material and the enzyme immobilization method. A bioinert, swollen PEG‐based hydrogel acted as a humidified substrate for a spot‐shaped, spatially defined GM coating, thereby preventing dehydration of the GM spots or unspecific interaction with enzyme substrates. GM hydrogels, known to be an appropriate host for enzymes,^[^ ^34^, ^45^ ^]^ were anchored to the PEG‐based hydrogel surface. The spots were supposed to serve as biocompatible entrapment for a biosensing component, the enzyme‐loaded TMV_Cys_. Additionally, the spatially defined coating should enable to incorporate two different enzymes into selectively reactive spots within a single sensor array in a straight‐forward way, to detect multiple analytes. In this configuration, coupling of the enzymes to TMV was expected to reduce their washout from the gel and thereby yield a reusable biosensor with a high amount of stably incorporated enzyme, as analyzed in proof‐of‐concept experiments as follows.

## 2 Results and Discussion

### 2.1 Surface Chemistry Requirements of the Hydrogel Substrate

**Scheme 2:**
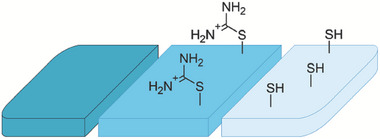
Schematic drawing of the different surface chemistries of the hydrogel substrates based on PEG. Left: Unmodified, non‐functionalized PEGDA. Middle: PEGDA functionalized with isothiouronium groups. Right: PEGDA functionalized with thiol groups.

In a first step toward creating a multi‐material combination as the basis of an all‐soft biosensor, we aimed to prepare spatially defined GM hydrogel coatings on a PEGDA hydrogel carrier (Scheme 1). PEGDA substrate layers were chosen due to their advantageous properties described in the introduction. GM spots were applied onto their surface as drops of a non‐cross‐linked GM solution which was cross‐linked by UV irradiation afterward. Therefore, the hydrogel substrate had to fulfill three requirements: First, it should not be wetted too well by the GM solution so that well‐defined spots can be formed. Second, it must be possible to cross‐link the GM on the hydrogel substrate while preventing dehydration of the spots. Third, the GM spots have to adhere to the PEGDA surface after cross‐linking and should remain anchored during prolonged submersion and washing periods. Consequently, PEGDA hydrogels with two alternative functionalities introduced into their surfaces were compared to a third plain (unmodified) preparation. The different surfaces displayed 1) non‐functionalized PEGDA, 2) isothiouronium groups, or 3) thiol groups (**Scheme** **2**). The isothiouronium groups were introduced by copolymerization of PEGDA with the monomer 2‐(11‐(acryloyloxy)‐undecyl)isothiouronium bromide (AUITB) (Figure S1, Supporting Information), thiols were generated by subsequent reduction of the isothiouronium groups, as described in our previous report (see also Experimental Section).^[^ ^46^ ^]^ Isothiouronium groups may interact with GM by ionic interactions, similar to the isothiouronium‐functional dye alcian blue that is well known to stain GM‐based materials.^[^ ^47^ ^]^ Thiol groups may react with methacrylate and methacrylamide groups in GM via a thiol‐Michael reaction to form covalent bonds between the hydrogel surface and the GM spots.^[^ ^48^ ^]^

Properties of the distinct PEGDA substrates with regard to their wetting behavior, spot cross‐linking, and surface adhesion will be described and discussed in the following two sections.

### 2.2 Wetting of the Hydrogel Surfaces by Gelatin Methacryloyl Solution

**Figure 1:**
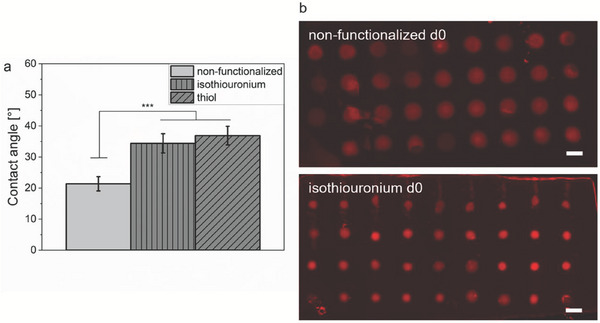
Investigation of the wetting behavior of the GM solution on differently functionalized surfaces of swollen PEG‐based hydrogels. a) Contact angle measurements of the GM solution on PEG‐based hydrogels. Hydrogels were either non‐functionalized, functionalized with isothiouronium groups, or functionalized with thiols (*n* = 3). Explanation of symbols: *** significant difference between non‐functionalized and functionalized hydrogels (*p* < 0.001). b) Exemplary fluorescence images on day 0 (d0) of GM spots on a non‐functionalized PEGDA hydrogel and an isothiouronium‐functionalized hydrogel (scale bar 1 mm). The spots were stained with fluorescent particles and cross‐linked before analysis with the method described in the next section.

The wetting behavior by GM solution on the surfaces of swollen PEG‐based hydrogels with the three different functionalities (Scheme 2) was first assessed by contact angle (CA) measurements with the sessile drop method, the results are shown in **Figure** **1**a. The composition of the GM solution was identical for all experiments, it contained 7.5 wt% GM with a photo initiator in phosphate‐buffered saline (PBS).

The CA of the GM solution was relatively low (21.4° ± 2.3°) for non‐functionalized hydrogels, while it was higher for both functionalized surfaces (34.4° ± 3.1° for isothiouronium‐functionalized and 36.9° ± 3.0° for thiol‐functionalized hydrogels). The difference between the non‐functionalized and the two functionalized hydrogels was highly significant (*p* < 0.001). The trend is similar to our previous report on water CA on non‐functionalized and isothiouronium‐functionalized PEGDA hydrogels and can be explained by the hydrophobic hydrocarbon chain of the AUITB monomer used for functionalization.^[^ ^46^ ^]^ However, the CA of the GM solutions was generally lower than that of water, presumably because of the presence of both salts and GM.

In order to further analyze the wetting behavior, arrays of GM spots on the PEGDA surfaces were generated by inkjet printing. This method was chosen because it allows to produce a high number of homogeneous spots in an automated and fast way for analysis. Inkjet printing appeared appropriate to generate spatially defined GM spot coatings on the hydrogels, as tested with a 10 pL drop volume and a predetermined spot diameter of 250 µm, as defined by the printing pattern. Both viscosity and surface tension of the GM solution at the printing temperature of 28 °C (Figure S3, Supporting Information) are in the range of other liquids that can be processed by inkjet printing, although the surface tension is slightly higher than typical for most inks.^[^ ^25^, ^49^, ^50^ ^]^ At the given temperature, the least nozzle clogging caused by solvent evaporation occurred, and drop formation was relatively stable. At lower GM concentrations, solutions were not viscous enough, and higher GM concentrations caused nozzle clogging. The number *Z*, a measure for inkjet printability, was determined by Equation (1), giving 11.96 ± 2.31. According to Fromm, usually satellite drops occur for a *Z* value > 10, which can influence the printing accuracy.^[^ ^51^ ^]^ Satellite drops were indeed observed during printing, however, they coalesced with the main drop in most cases so that a deterioration of the printed pattern was not recognized with the bare eye. Our results are in good agreement with the report by Hoch et al. who processed solutions of a similar GM by inkjet printing.^[^ ^25^ ^]^ They reported a reproducible printing process of an ink containing 5% GM at 25 and 37 °C. For 10% and 15% GM the printing process at 37 °C could not be restarted after a printing pause due to clogging of the nozzle.^[^ ^25^ ^]^ It should be noted that to investigate the GM spots after printing visually, they needed to be stained. To this end, fluorescent nanoparticles were mixed into the GM solution. No influence of the staining on the printability was observed.

For inkjet‐printed GM spot arrays on non‐functionalized PEGDA hydrogels (Figure 1b, upper row), an average surface area *A* per spot of 0.725 mm^2^ ± 0.139 mm^2^ was found. Due to the low CA (Figure 1a), the printed solution spread rather well on the PEGDA surface before curing, resulting in rather blurry spots. The CA suggested that the GM solution would spread less on the functionalized hydrogels, and thus these were expected to better support spatially well‐defined spots. Indeed, on isothiouronium‐functional hydrogels (Figure 1b, lower row) an average *A* of 0.316 mm^2^ ± 0.174 mm^2^ was observed, and on thiol‐functional hydrogels of 0.343 mm^2^ ± 0.151 mm^2^. Statistically, the *A* values between non‐functionalized and functionalized hydrogels were different (*p* < 0.05), in agreement with the CA results discussed before.

The combined results show that the functionalized hydrogels are more appropriate surfaces for a precisely defined GM coating pattern with a smaller surface area of the spots, and hence for a biosensing platform. Between the two functionalized surfaces, no difference was found, so both should be equally suitable as sensor substrates.

### 2.3 Cross‐Linking and Stability of Gelatin Methacryloyl Spots

**Figure 2:**
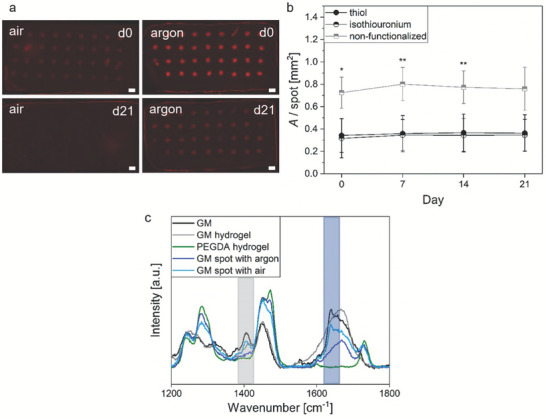
Evaluation of the cross‐linking efficiency of GM spots. a) Exemplary fluorescence images of GM spots were either cross‐linked under ambient conditions, left panel, or in a humidified argon atmosphere, right panel, on a thiol‐functionalized hydrogel monitored over 21 days, scale bar 1 mm. b) Determined surface areas *A* of the GM spots, which were cross‐linked under argon atmosphere, on days 0, 7, 14, and 21 (*n* = 3; non‐functionalized hydrogels day 21 *n* = 2). Explanation of symbols: * or ** significant difference between non‐functionalized and functionalized hydrogels (*p* < 0.05 or *p* < 0.01). c) Raman spectra of pure GM, a GM hydrogel, a PEGDA hydrogel, a GM spot that was cross‐linked under argon atmosphere, and a GM spot cross‐linked under ambient conditions. The gray area highlights the band of the C═C in‐plane scissoring vibration and the blue area highlights the band of the C═C stretching vibration. Due to the low thickness of the GM spots (≈2 µm, estimated from the Raman microscopic data), it was not possible to record pure spectra without bands of the PEGDA support. Therefore, a PEGDA hydrogel was measured as a reference.

Apart from appropriate wetting of the hydrogel substrate surface, the dispensed GM solution has to be sufficiently cross‐linked in order to physically entrap the TMV‐coupled sensing component (Scheme 1) stably over prolonged submersion and washing times. Cross‐linking efficiency was assessed by the integration of fluorescent polystyrene nanoparticles with an average diameter of 100 nm, which would be physically entrapped inside a properly cross‐linked GM hydrogel due to its estimated smaller mesh size, and as a consequence cannot be washed out.^[^ ^45^ ^]^ The spot‐coated hydrogels were immersed in water after UV illumination and evaluated by fluorescence microscopy once a week over a period of 21 days. In preliminary experiments, we faced severe problems with successful cross‐linking with UV light when irradiating the liquid GM spots on the swollen hydrogel substrates in contact with the ambient atmosphere. When immersed in water and agitated gently after illumination, the GM spots were washed away over time so that only a diffuse nanoparticle fluorescence was detectable after 21 days (**Figure** **2**a, left panel). This suggested that the 100 nm particles were washed out because the spots were not cross‐linked sufficiently. By contrast, GM solution with the same composition could be cured without issues in the same mold covered with a quartz glass pane but purged with humidified argon (Figure 2a, right panel). We hypothesized that the difference in curing behavior was either caused by premature drying of the spots or oxygen inhibition of the free radical cross‐linking process due to the low thickness of the spots. Indeed, photopolymerization with initiators like the used lithium phenyl‐2,4,6‐trimethylbenzoylphosphinate (LAP) is known to be sensitive to oxygen.^[^ ^52^, ^53^, ^54^, ^55^ ^]^ Accordingly, the GM spots cross‐linked in the humidified argon atmosphere remained stable over at least 3 weeks, as also evidenced by analyzing the surface area *A* of the spots. In fact, *A* remained virtually unchanged for all three tested surface variations of the PEGDA hydrogel substrates over the complete period of the experiments, showing that the combination of humidification and oxygen reduction promotes cross‐linking (Figure 2b). The analysis of *A* demonstrates that the main effect of surface functionalization is adapting the surface wetting. The bond between the cross‐linked GM spots and the hydrogel substrate surface was thus strong enough for all PEGDA hydrogel variants including the non‐functionalized one.

Apart from the indirect evidence of cross‐linking by way of nanoparticle entrapment, as described above, the cross‐linking was directly studied by observing the double bond‐related signals in Raman spectra of acrylic groups in GM. Raman spectra were measured of pure GM, a GM hydrogel cross‐linked in a quartz glass‐covered mold (i.e., without air contact), a cross‐linked PEGDA hydrogel, GM spots cross‐linked under argon atmosphere, and GM spots cross‐linked in contact with ambient air during the irradiation, respectively. As shown in Figure 2c, the typical band of the C═C stretching vibration between 1620 and 1680 cm^−1^ as well as the band of the C═C in‐plane scissoring vibration between 1390 and 1420 cm^−1^ was present when the GM spots were irradiated under ambient conditions, that is, in the absence of an argon atmosphere, similar to the spectrum of the pure GM.^[^ ^56^, ^57^ ^]^ In the presence of argon, the double bond‐related bands were similarly low like for the reference GM hydrogel cross‐linked in a mold. These observations are in accordance with no success in cross‐linking via free radical cross‐linking chemistry involved during GM spot irradiation in contact with air (see Scheme S1, Supporting Information, for schematic representation of cross‐linking chemistry), while under argon atmosphere, the acrylic double bonds of the methacryl‐modified gelatin GM were converted to cross‐links.

The exact mechanism of GM spot surface anchoring remains speculative because it is not possible to extract chemical information specifically for the interface between PEG‐based hydrogel and GM spots from the Raman spectra; however, we attribute the successful surface anchoring to sufficient entanglements between the polymer chains, and to hydrogen bonds between GM and the PEG backbone. Another possibility is the participation of remaining acrylate groups within the PEG‐based hydrogels in the cross‐linking of GM since it was reported that not all acrylate groups are consumed during radical cross‐linking of PEGDA.^[^ ^58^, ^59^ ^]^ In conclusion, the two types of PEGDA surface modifications promote precise spatial arrangements of small GM spots but are not essential for their stable anchoring.

Taken together, the Raman spectra and the fluorescence images consistently revealed that the cross‐linking of the GM spots was successful in a humidified argon atmosphere on all three tested PEGDA surfaces, and also, GM spot anchoring worked in all cases. However, spots were smaller and more defined on the two types of chemically functionalized surfaces, without a difference between isothiouronium and thiol modification. Therefore, the isothiouronium‐functionalized surfaces were used further as a hydrogel substrate due to the smaller effort for sample preparation.

### 2.4 Synthesis of Enzyme‐Loaded TMV_Cys_

**Figure 3:**
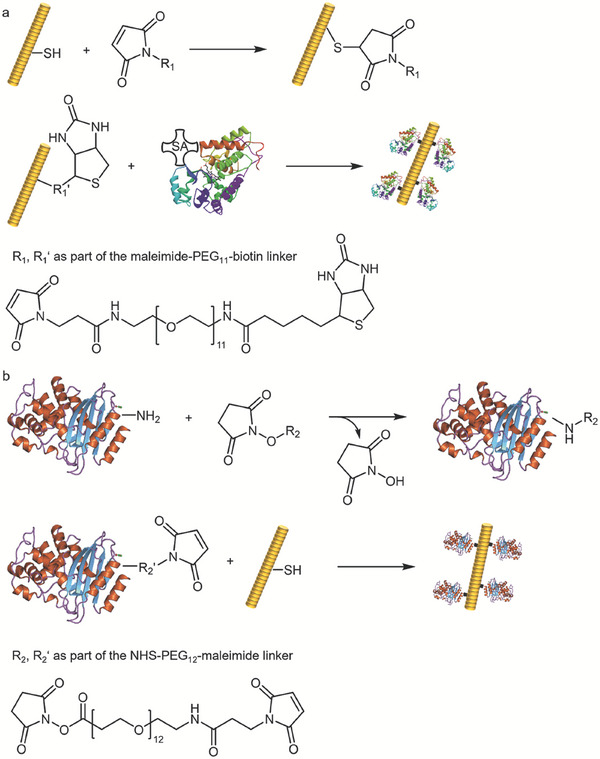
Coupling of cysteine‐modified TMV nanorods to linkers and enzymes, visualized for a single or few out of around 2000 docking sites only for reasons of clarity. a) TMV_Cys_ (yellow rod) was coupled to the maleimide‐PEG_11_‐biotin linker via its thiol groups (SH). In the next step, SA‐HRP was bound to the linkers due to the strong affinity for biotin. b) The enzyme penicillinase was coupled to NHS‐PEG_12_‐maleimide linkers via its surface‐exposed primary amine groups (NH_2_), generating NHS leaving groups. Subsequently, TMV_Cys_ was bound to the maleimide functionality of the enzyme‐coupled linker via the thiol groups exposed to the CP subunits.

After the successful preparation of the GM spots on the PEG‐based hydrogel, the analyte receptor/detector component of the biosensor was prepared, that is, the enzyme‐loaded TMV nanoparticles enabling colorimetric read‐out (Scheme 1). We chose to integrate HRP or penicillinase (Pen) into our spatially defined coatings in order to detect the enzyme‐catalyzed reactions either of hydrogen peroxide or β‐lactam antibiotics, respectively, with appropriate chromogenic substrates. TMV‐conjugated and free enzymes were applied in parallel tests. HRP was used as its conjugate with streptavidin (SA‐HRP) and was bound to TMV_Cys_ via a biotin linker, which was covalently coupled to the virus via its maleimide functionality (**Figure** **3**a), as described by Koch et al.^[^ ^39^ ^]^ A coupling efficiency of TMV_Cys_ to the biotin linker of 93% was determined (Figure S4, Supporting Information). SA‐HRP was coupled to TMV nanocarrier rods by bioaffinity binding via biotin‐streptavidin interaction. Offering 2,2′‐azino‐bis(3‐ethylbenzothiazoline‐6‐sulfonic acid) (ABTS) substrate solution to this reaction product resulted in the development of a green stain in the solution (results not shown), indicating the successful formation of the TMV‐HRP conjugate and that the enzyme functionality was retained. Pen was conjugated to TMV_Cys_ by first reacting the enzyme with the NHS ester of a heterobifunctional linker via accessible lysine amino groups of the enzymes. Subsequently, the maleimide‐functional other end of the linker was reacted with TMV_Cys_ via a thiol‐Michael reaction, yielding the TMV‐Pen conjugate (Figure 3b). For Pen, the enzyme activity was verified by the yellowish nitrocefin substrate turning red upon hydrolysis of its β‐lactam ring.

Apart from the successful coupling of the enzymes, it was equally important to us that the TMV particle structure remained intact after the synthetic procedures so that they would be physically entrapped when integrated into GM hydrogel spots (see also the following section). Therefore, the TMV‐HRP and TMV‐Pen conjugates were analyzed by transmission electron microscopy (TEM). Although under the conditions applied the enzyme decoration could not be detected around TMV‐HRP or TMV‐Pen, respectively, as visualized by Koch et al. earlier,^[^ ^39^, ^40^ ^]^ the rod‐like structure of TMV with its typical 300 nm length, elongated oligomeric head‐to‐tail aggregates and a few shortened particles confirmed the structural integrity of TMV‐nano‐adaptors, rendering them suitable for entrapment within a cross‐linked hydrogel matrix (Figure S4, Supporting Information).

### 2.5 Assembly of the All‐Soft Biosensor

**Figure 4:**
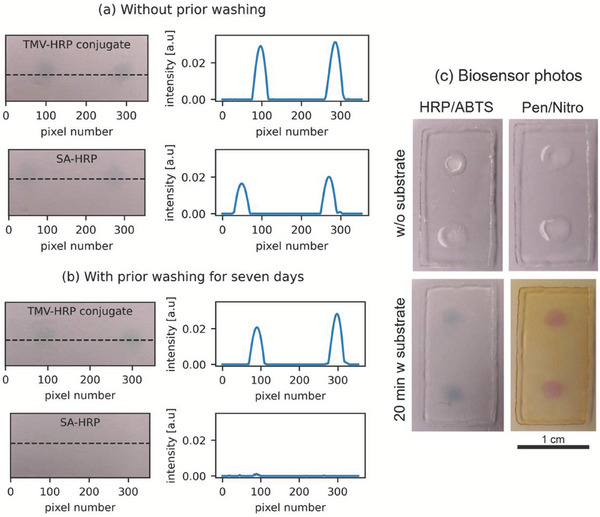
Analysis of the sensor enzyme activities in GM spots anchored on a hydrogel substrate. a) When offering ABTS for 20 min directly after sensor preparation, that is, without any washing steps, the enzymatic activity of HRP was found both for TMV‐conjugated HRP (top left) and unconjugated SA‐HRP (bottom left), as evidenced by the green color of the spots. Right next to the photos, the respective color intensity along the dashed line in the photos is plotted. b) After extended washing of the samples, only the samples containing the TMV‐conjugated HRP showed enzyme activity, indicating a stable immobilization of the TMV‐displayed enzyme in the GM spots. Unbound SA‐HRP was removed completely from the samples due to diffusion through the hydrogel meshes. c) Representative photos of GM spots on PEG‐based hydrogels without (w/o) the substrate solution (top row), and after 20 min with (w) substrate solution (bottom row). Left: GM spots with TMV‐conjugated HRP and the addition of ABTS solution (HRP/ABTS), which turned the spots green. Right: GM spots with TMV‐conjugated Pen and addition of yellow nitrocefin solution (Pen/Nitro), which turned the spots red and the PEG‐based hydrogel yellow. Scale bar 1 cm.

In the final step, the three soft material components, that is, the inert, humidified PEGDA hydrogel support substrate, the biobased GM spots, and the enzyme‐loaded TMV particles were combined in a single assembly to demonstrate a biosensor functionality. For this purpose, TMV‐HRP was suspended in the GM solution and the suspension was pipetted on the PEG‐based hydrogels manually to obtain larger spots, simplifying the detection of a color reaction. As a control, free SA‐HRP devoid of TMV was added to the solution instead of TMV‐HRP before cross‐linking. After cross‐linking of the enzyme‐containing GM spots, the hydrogels were immersed in substrate solution either immediately (without prior washing), or after washing them for 1 week under agitation. Color formation was evaluated after a reaction time of 20 min (see **Figure** **4**). After an additional 5 min, the substrate solution was removed and used for absorption measurements indicative of the HRP‐catalyzed reaction of hydrogen peroxide with ABTS (see **Table** **1** and corresponding discussion below).

**Table 1:** Ratio of absorption values of ABTS solution after reaction for 25 min with samples and absorption values of non‐used solution.

Sample	Non‐washed	Washed
TMV‐HRP	1.311 ± 0.076	1.022 ± 0.010
SA‐HRP	1.500 ± 0.057	0.994 ± 0.006

Samples were the fully assembled soft sensor, that is, PEG‐based hydrogels with GM spots containing either TMV‐HRP conjugate or unbound SA‐HRP. Samples were non‐washed or washed before the reaction. Values are displayed as mean ± SD (*n* = 3).

We found that the GM spots containing the TMV‐HRP conjugate started colorless and developed a dark green color after adding the ABTS substrate solution to the hydrogels (see photos in Figure 4c, for comparison see photos after 0 min reaction time in Figure S5, Supporting Information). This was the case for both unwashed and washed samples. For non‐washed hydrogels, the solution surrounding the spots turned green as well. Higher green intensities of the spots were detected for the non‐washed samples compared to the washed ones, as evident when evaluating the color intensity profiles (Figure 4a,b). By contrast, for GM spots containing unbound SA‐HRP, the spots and the surrounding solution turned green only when no washing procedure was applied. The spots, as well as the solution, remained completely colorless if the hydrogels were washed, as also shown by the corresponding intensity profiles (Figure 4a,b and Figure S5, Supporting Information). The application of TMV_Cys_‐bound SA‐HRP therefore retained the enzymatic activity within the GM hydrogel by preventing wash‐out of the bioactive molecules. Enzyme‐loaded TMV carrier rods obviously did not diffuse out of the GM hydrogel with its estimated mesh size of ≈10–30 nm^[^ ^45^ ^]^ to a significant extent. This is in line with the findings of Claaßen et al.^[^ ^45^ ^]^ who showed that SA‐HRP may diffuse into a GM hydrogel and bind a biotin‐functional linker coupled to the hydrogel. In conclusion, loss of free SA‐HRP from GM spots through diffusion into the surrounding solution needs to be prevented by immobilizing the enzyme, for which its coupling to TMV carrier scaffolds retained inside the hydrogel pores due to its dimensions appears as an efficient and versatile strategy.

In order to compare the loss of enzyme activity within spots with or without TMV carrier rods upon washing in more detail, absorption measurements at 405 nm were carried out with the ABTS solution that was in contact with the hydrogels, and with non‐used (fresh) solution as reference. In case of an enzymatic activity outside the GM hydrogel spots, the chromogenic reaction would increase the absorbance resulting in ratios above 1, while in the case of no color reaction outside the spots, the ratio should stay equal to 1. The ratios of the two absorption values are collected in Table 1 (numeric absorption values are displayed in Table S2, Supporting Information). The results were in accordance with the visual impression described above: An absorption increase was detected for non‐washed samples directly after their preparation both for TMV‐HRP‐ and SA‐HRP‐containing samples. This indicates that some SA‐HRP has leached out of the GM hydrogel spots in both cases, moving enzyme activity into the solution. Nevertheless, according to the change in absorbance ratios, leaching from unwashed spots was considerably less if SA‐HRP was immobilized on TMV. For TMV‐HRP samples, we interpret the leaching enzyme activity as a result of incomplete binding of SA‐HRP to TMV. Since the diffusion of TMV‐HRP out of the gel matrix is very unlikely due to the size of the complexes compared to the mesh size of the gel, and the SA‐biotin‐binding affinity is very high, we conclude that some unbound SA‐HRP existed as a by‐product in the TMV‐enzyme preparation. The wash‐out of such free SA‐HRP from GM spots most likely resulted in the signal drop observed in the corresponding spots after 7 days of washing (Figure 4). After extensive washing, none of the biosensors showed any color reaction outside the GM hydrogel spots (Table 1, absorbance ratios of incubated substrate solution vs non‐incubated solution close to 1). Biosensors with GM hydrogel spots harboring free SA‐HRP lost their signals completely, both within the spots and in the surroundings after washing. In comparison, washed biosensors with TMV‐HRP conjugates showed a color reaction limited exclusively to the GM hydrogel spot (see Figure 4). On the one hand, this highlights the need for properly washing the biosensors presented here prior to operation, in order to remove potentially unbound enzyme from the spots; on the other hand, and most importantly, this demonstrates the capability of TMV to retain enzyme activity within GM hydrogels.

Another finding is that the enzyme HRP, coupled to the virus particles or not, maintained activity after UV irradiation. Our group reported earlier that the protein vascular endothelial growth factor (VEGF) incorporated into GM hydrogels was impaired by the free radical cross‐linking process.^[^ ^60^ ^]^ Hence, it is not foreseeable if proteins maintain their function if integrated in a hydrogel precursor solution before photo cross‐linking. Wang et al. also showed that HRP in the buffer was less active or completely inactivated after pulsed light treatment due to aggregation and changes in the protein structure.^[^ ^61^ ^]^ It is thus likely that the specific process conditions applied did not affect HRP functionality to a considerable extent, although our analyses would not detect a minor impairment of the activity of the enzyme.

After demonstrating that the soft biosensor worked with TMV‐HRP, Pen was used to test a second enzyme in the same hydrogel layout, and concomitantly also an alternative coupling method on the viral nanocarrier. Pen has a molecular weight of ≈28 kDa and is commercially available as a mixture of two *ß*‐lactamases.^[^ ^40^ ^]^ Since SA‐HRP has a molecular weight of at least 90 kDa and is therefore much bigger than Pen, it was assumed that uncoupled Pen would have even higher mobility in GM hydrogel spots, resulting in its wash‐out during 1 week as well. Therefore, only TMV‐exposed Pen was analyzed for both its antibiotics detection capability and reusability inside GM spots. Different from earlier applications in TMV‐assisted biosensors,^[^ ^40^ ^]^ the enzyme was bound covalently to TMV via PEG_12_‐linkers before its incorporation into the GM spots as described above. When these hydrogels were immersed in yellow nitrocefin substrate solution, the spots on both washed and non‐washed samples turned red without any such color change of the surrounding solution. The transparent hydrogel supports soaked by the substrate solution adopted the yellow educt color (Figure 4c right).

These results collectively show that GM spots on PEG‐based hydrogels were well‐suited to encapsulate and host enzymes installed on TMV. Nanocarrier retention inside the gel was in accordance with results from a previous study on TMV incorporated into PEG hydrogels,^[^ ^42^ ^]^ and contrasted with our findings for the diffusion of free enzymes into the surrounding solution. HRP and Pen both retained their activity after incorporation into the hydrogel as indicated by the chromogenic conversion of their substrates. Hence, the combination of the two different types of enzyme‐containing GM spots in a single sensor array was tested. PEG‐based hydrogels were coated with one spot containing TMV‐Pen conjugate and a second one with TMV‐HRP conjugate. Following the addition of nitrocefin solution, the TMV‐Pen spot stained red indicating Pen activity. After replacing the nitrocefin with ABTS solution, a slightly green stain developed in the TMV‐HRP spot, which, however, appeared much less intense than in the case of ABTS addition without prior application of nitrocefin. Presumably, the color reaction was less visible due to the yellow stain of the PEG support, or the activity of HRP was impaired by residual nitrocefin. If the chromogenic substrates were applied in inverted order or simultaneously, that is, ABTS first and nitrocefin second, or in a mixture, both colors developed as well, with, however, red stain also forming on the HRP‐loaded spot after nitrocefin addition. This side reaction masked the green color either after its occurrence or obviated its reliable detection (results not shown). A different read‐out system will thus be necessary for real applications.

The enzyme HRP was chosen due to its good commercial availability and easy detection of its activity via a color reaction. The application of Pen demonstrated that the system is appropriate to work in biomedically relevant biosensors and that this sensor layout could be transferred to other enzymes which may enable environmental and food monitoring as well. Since the enzymes remained active and were stably immobilized on TMV particles in the sensor matrix for at least 1 week, the system showed potential to be reused with consecutive samples after the wash‐out of the preceding analyte solution. The storage stability and shelf‐life of the specimens were assayed after keeping the air‐dried biosensors after the initial tests in a refrigerator for ≈22 months. They were re‐hydrated and extensively washed before fresh substrate solutions were added. Whereas for TMV‐HRP‐containing biosensors, no enzyme activity was detectable anymore, TMV‐Pen‐equipped ones still induced a visible color reaction in the GM hydrogel spots, indicating preserved Pen activity and thus antibiotics sensing capacity (see Figure S8, Supporting Information). With the spatially well‐defined GM spot array and a known number of docking sites exposed on the TMV nanocarriers, it will be feasible to integrate a defined number of enzyme molecules per spot and to combine distinct enzymes on one biosensor platform via GM formulations with different nanoparticle‐enzyme conjugates. TMV has already been successfully applied in various sensor layouts^[^ ^39^, ^40^, ^41^, ^62^, ^63^, ^64^, ^65^, ^66^, ^67^, ^68^, ^69^, ^70^ ^]^ and was shown to stabilize distinct enzymes including penicillinase in label‐free electrochemical field‐effect biosensors that retained their performance for at least a year of repetitive use.^[^ ^41^ ^]^ This points to the future potential of the combination of plant viral bioscaffolds with hydrogel soft biosensors, for example, as sensor domains in 3D cell cultures grown for tissue replacement, in implanted online sensors, or for in situ detection purposes for process monitoring in biotechnological or food fabrication fermenters. In this study, the functionality of the all‐soft qualitative biosensor made of a PEG hydrogel as a substrate and GM spots as a sensor component was successfully demonstrated with the two enzymes HRP and Pen.

## 3 Conclusions

In this contribution, we show that GM spots bound to a PEG‐based hydrogel surface can serve as a versatile, qualitative soft biosensor array. Contact angle measurements and inkjet printing of GM solution on PEG hydrogels revealed that both isothiouronium and thiol functionalization of the PEG reduce the spreading of the GM solution on the surface and thereby help to obtain more precisely defined spot patterns than on unmodified PEG. The GM spots were successfully UV‐cross‐linked under a humidified argon atmosphere. Groups of the enzymes HRP or Pen were immobilized on cysteine‐exposing engineered TMV carrier rods, which enabled to stably incorporate and capture the active enzymes in the GM hydrogel matrix for at least 1 week, whereas free enzymes were washed out. In the case of Pen, the reusability of the detector array was even demonstrated after almost 2 years of dry storage in the cold followed by rehydration. In conclusion, the hierarchically constructed soft biosensor made of a functionalized PEG‐based hydrogel coated with GM spots containing virus‐scaffolded enzymes is a promising approach for the detection of various substrates and might allow the fabrication of reusable sensors with high storage stability. Moreover, spatially defined spot array coatings on a hydrogel platform could allow the simultaneous use of multiple enzymes for the detection of multiple analytes. The sensitivity and repeated use of the biosensor may be further evaluated and the combination of different TMV‐nano‐scaffolded enzymes in selectively reactive GM spots may be developed into bi‐ or multifunctional arrays for sensing distinct analytes in complex mixtures or serially applied samples in future work.

## 4 Experimental Section

### Chemicals and Materials

The following chemicals were purchased from Merck KGaA (Darmstadt, Germany): ABTS liquid substrate system, ammonium hydroxide (NH_4_OH) ≈28–30% (m/m), acryloyl chloride ≥97%, deuterated chloroform (CDCl_3_) 99.8%, dimethylformamide (DMF), ethanol (EtOH) absolute for analysis, hydrogen peroxide (H_2_O_2_) 30% (m/m), hydroquinone, 2‐hydroxy‐4′‐(2‐hydroxyethoxy)‐2‐methylpropiophenone (Irgacure 2959), lithium phenyl‐2,4,6‐trimethylbenzoylphosphinate (LAP), methacrylic anhydride (MAAnh), nitrocefin, penicillinase from *Bacillus cereus* ((1500–3000) units mg^−1^ protein), PEGDA *M*_n_ ≈ 700 g mol^−1^, potassium chloride (KCl) for analysis, sodium hydroxide (NaOH), sodium metabisulfite (Na_2_S_2_O_5_), sodium phosphate monobasic monohydrate (NaH_2_PO_4_ * H_2_O), sodium phosphate dibasic dihydrate (Na_2_HPO_4_ * 2 H_2_O), and triethylamine ≥99.5%. Chloroform ≥99.8% and dichloromethane (DCM) ≥99.8% were purchased from Honeywell (Offenbach, Germany). Technical acetone and isopropanol were purchased from Brenntag GmbH (Essen, Germany). Dimethyl sulfoxide (DMSO) for synthesis and tetrahydrofuran (THF) for HPLC were purchased from ChemSolute (Th.Geyer, Renningen, Germany). 11‐bromo‐1‐undecanol was purchased from TCI GmbH (Eschborn, Germany). Ethylene‐diamine‐tetraacetic acid (EDTA), sodium bicarbonate (NaHCO_3_), sodium chloride (NaCl), disodium hydrogen phosphate (Na_2_HPO_4_), 3‐(*N*‐morpholino)propanesulfonic acid (MOPS), potassium dihydrogenphosphate (KH_2_PO_4_), chemicals for SDS‐PAGEs, thiomersal, and tris (2‐carboxyethyl) phosphine hydrochloride (TCEP), were purchased from Carl Roth GmbH + Co. KG (Karlsruhe, Germany). Maleimide‐PEG_11_‐biotin, Pierce SM(PEG)_12_ (maleimide‐PEG_12_‐NHS), and PageRuler Prestained were purchased from Thermo Fisher Scientific GmbH (Dreieich, Germany). Other reagents were purchased from the following companies (given in parentheses): deuterated dimethyl sulfoxide (DMSO‐d_6_) 99.8% (Deutero, Kastellaun, Germany), ethyl acetate for HPLC (VWR Chemicals, Darmstadt, Germany), fluorescence‐labeled particles micromer‐redF, plain, 100 nm (micromod Partikeltechnologie GmbH, Rostock, Germany), hydrochloric acid (HCl) 37% (m/m) (Häberle Labortechnik GmbH & Co, Lonsee‐Ettlenschieß, Germany), GE low molecular weight Marker (Amersham, Buckinghamshire, UK), magnesium sulfate 99% anhydrous (abcr GmbH, Karlsruhe, Germany), nitrogen gas (Air Liquide, Düsseldorf, Germany), potassium hydroxide (KOH) (AppliChem GmbH, Darmstadt, Germany), streptavidin‐coupled horseradish peroxidase (SA‐HRP, 1 mg mL^−1^, SDT GmbH, Baesweiler, Germany), and thiourea (Alpha Aesar, Kandel, Germany). Gelatin (type B, limed, bovine bone, 232 bloom) was kindly provided by Gelita, Eberbach, Germany.

Acryloyl chloride was distilled before use. PBS pH 7.4 was freshly prepared with 137 mm NaCl, 2.7 mm KCl, 1.5 mm KH_2_PO_4_, and 8.1 mm Na_2_HPO_4_ * 2 H_2_O in deionized water. 0.1 m Phosphate buffer pH 7 was freshly prepared with 0.05 m Na_2_HPO_4_ * 2 H_2_O and 0.05 m NaH_2_PO_4_ * H_2_O.

10 mm sodium‐potassium‐phosphate buffer (SPP) pH 7.2 was freshly prepared from autoclaved 0.5 m SPP stock solution which was prepared by adjusting the pH of 400 mL 0.5 m KH_2_PO_4_ solution by adding 0.5 m Na_2_HPO_4_ solution to a final pH‐value of 7. After diluting the SPP‐stock 1:50 with deionized water, the resulting 10 mm SPP was autoclaved.

Silicon wafers, type p/bor, were purchased from Si‐Mat Silicon Materials (Kaufering, Germany).

### Preparation of PEG‐Based Hydrogels

The synthesis of the monomer AUITB containing an isothiouronium group was performed as described earlier.^[^ ^46^ ^]^ AUITB was synthesized in a two‐step reaction. In brief, 11‐bromoundecyl acrylate was synthesized by acrylation of 11‐bromo‐1‐undecanol with acryloyl chloride. In the second step, AUITB was obtained by nucleophilic substitution with thiourea.

PEG‐based hydrogels were prepared as described before.^[^ ^46^ ^]^ Non‐functionalized hydrogels were prepared by dissolving Irgacure 2959 at a concentration of 0.5 wt% in PEGDA under the protection of light. For a functionalization of the hydrogels, first AUITB was dissolved in PEGDA by heating, followed by dissolution of Irgacure 2959 under the protection of light, so that concentrations of 2 wt% (AUITB) and 0.5 wt% (Irgacure 2959) were reached. Silicon wafers were cleaned and activated with H_2_O_2_ and NH_4_OH in a volume ratio of 2:3 at 70 °C. The hydrogel precursor solution was poured inside a silicone frame (500 µm height) on the silicon wafers. The mold was closed with a quartz glass pane and left at room temperature for 5 h under the protection of light. Afterward, the solution was polymerized by UV‐irradiation for 7.5 min (radiation intensity of 50 mW cm^−2^, spectral range >300 nm, with an emission maximum around ≈365 nm, sol2, Dr. Hönle AG). The hydrogel surface orientated toward the activated silicon wafer was used further on.

Hydrogels were treated with sodium metabisulfite (Na_2_S_2_O_5_) to reduce the isothiouronium groups to thiols, generating the third hydrogel surface. For this purpose, the non‐functionalized and isothiouronium‐functionalized PEGDA hydrogels were swollen in deionized water for at least 16 h and were punched in the size of 1 cm × 2 cm. Non‐treated and non‐functionalized samples were immersed in 5 mL of deionized water at room temperature. Non‐functionalized control samples and isothiouronium‐functionalized samples were treated with 5 mL 1 m Na_2_S_2_O_5_ for 5 h at 60 °C. After several washing steps with PBS, all hydrogels were washed with deionized water and were kept in water until further use.

### Synthesis of Gelatin Methacryloyl (GM10)

GM was synthesized as described before.^[^ ^71^ ^]^ In brief, 25.06 g gelatin were dissolved in 250 mL deionized water at 40 °C and the pH was adjusted to 7.25 with an automatic titration device. 13.52 g methacrylic anhydride (MAAnh), corresponding to a tenfold molar excess relative to the free amino groups in gelatin,^[^ ^72^ ^]^ were added slowly. The reaction mixture was stirred for 5 h. Afterward, the pH was adjusted to 9.5 and the mixture was filtrated and kept at 4 °C for 2 days. After adjusting the pH to 9.5 again, the mixture was filtrated with a bottle top filter and dialyzed with deionized water for 4 days at room temperature. The pH was adjusted to 8.5 and the solution was lyophilized. The degree of methacrylation (DM) was determined with ^1^H NMR, as described by Claaßen et al.^[^ ^71^ ^]^ GM_10_ batches with a DM of (0.809 ± 0.015), (0.869 ± 0.034), (0.965 ± 0.003), 1.014, (1.043 ± 0.061), (1.072 ± 0.116), and (1.137 ± 0.023) mmol g^−1^ were used (NMR spectrum in Figure S2, Supporting Information). Since GM_10_ was the only GM derivative used, it is otherwise called GM.

### Preparation of the GM Solution and its Characterization

GM and the photo initiator LAP were dissolved in PBS to prepare a solution with 7.5 wt% GM and 0.7 wt% LAP, relative to the biopolymer mass. The GM solution was protected from light.

The wetting behavior of the GM solution was investigated by CA measurements on swollen non‐functionalized and functionalized PEGDA hydrogels with a Teflon‐lined cannula. For this purpose, the sessile drop method was used and the CA of a 2 µL drop was determined at *t* = 1 s with video analysis (DataPhysics Instruments GmbH, OCA 40). The viscosity of the GM solution was examined with a rheometer (MCR 301, Anton Paar GmbH) with the measuring cone CP40. The measuring plate and cone were heated up to 28 °C because this temperature was used for inkjet printing. 1.2 mL of the solution was poured in the measuring gap and the viscosity was measured at a shear rate between 1 and 3000 s^−1^. The surface tension of the GM solution was determined with a bubble pressure tensiometer (BP50, Krüss GmbH). The solution was analyzed at 28 °C and the surface age was determined between 15 and 16 000 ms. To characterize the drop formation during the inkjet printing process, the number *Z* ^[^ ^51^ ^]^ was calculated with the following equations

(1) Z=1Oh=ReWe=σρdη

with Oh being the Ohnesorge number, Re the Reynolds number, and We the Weber number. *d* is the diameter of the print heads with 21.5 µm, the viscosity *η* was used at a shear rate of 208 s^−1^, and the surface tension *σ* at the smallest surface age of 15 ms. For the density *ρ* of the GM solution, an average value of 1.01 g mL^−1^ was used.

(2) Re=ρϑdη

(3) $$\mathrm{We}\;=\;\frac{\rho \vartheta^{2}d}{\sigma }$$

### Inkjet Printing and Analysis of Fluorescence‐Labeled GM Spots

Spatially defined bioactive coatings on the hydrogel surfaces were generated either by pipetting or with an inkjet printer (Dimatix DMP 2850, Fujifilm), depending on the desired quantity and volume of the spots. For printing, a print head (DMC‐11610) with a drop volume of 10 pL was used. A pattern with 4 × 9 spots was chosen to investigate the spot size and shape on the different hydrogel surfaces by printing with a labeled GM solution at a frequency of 2 kHz. For this purpose, 30 wt% of a solution containing 10 mg mL^−1^ fluorescence‐labeled polystyrene particles in water (redF, 100 nm) was added, leading to a concentration of 0.3 wt% of the particles in the solution. The amount of PBS was adjusted, respectively. The printer was protected from light to avoid polymerization and bleaching of the GM solution during the printing process. The spots with a predefined diameter of 250 µm and 5080 dpi were printed on the swollen hydrogels. The nozzle was heated up to 28 °C to avoid clogging. After printing, the coated hydrogels were UV‐irradiated (Hartmann.gs, UV‐H 225, Light Box Nr. 0191, 10.4 mW cm^−2^) for 2.5 min under argon atmosphere to reduce the oxygen concentration. For this purpose, an aluminum mold was constructed, which was purged with humidified argon, and the samples were put inside. Afterward, the hydrogels were kept in deionized water under the protection of light. At specific time points, the printed pattern was observed with a fluorescence microscope (BZ‐9000, Keyence) with 2× magnification by using the merge function. The determination of the surface area (*A*) of the spots was performed with self‐implemented Python scripts and Fiji.^[^ ^73^ ^]^ To distinguish between the back and foreground, a threshold for the intensity was used with the Otsu method. The results for the treated non‐functionalized control samples are displayed in Table S2, Supporting Information. The surface area *A* of the spots was calculated with the following equation, with 34 596 px mm^−2^ being the scaling of the images

(4) $$A\;\mathrm{per}\;\mathrm{spot}\;\;\left[{\mathrm{mm}}^{2}\right]=\;\frac{\mathrm{Number}\;\mathrm{of}\;\mathrm{pixels}\;\mathrm{per}\;\mathrm{spot}\;\left[\mathrm{px}\right]}{34596\;\left[\frac{\mathrm{px}}{{\mathrm{mm}}^{2}}\right]}$$

### Raman Spectroscopy

PEGDA hydrogels with printed 4 × 9 GM spots without the addition of the redF nanoparticles were evaluated with Raman microscopy (inVia Raman microscope, Qontor, Renishaw plc) to validate the cross‐linking of the GM spots. Raman spectra were recorded with a 100× microscope lens (Leica N PLAN EPI, WD 0.27 mm, NA 0.85) and a laser power of 100% with the 532 nm laser. After printing and washing in deionized water, the imprinted gels were dried under reduced pressure at 60 °C for 24 h (VDL 53, Binder GmbH). As a reference, a conventional prepared GM hydrogel was measured. It was made of the same solution, which was poured into an aluminum mold (1 mm height, 30 mm diameter). The mold was closed with a quartz glass pane and the solution was cross‐linked by UV‐irradiation for 2.5 min with the same intensity. All spectra were normalized before analysis.

### Coupling of TMV_Cys_ to a Maleimide‐PEG11‐Biotin Linker

The cysteine mutant of the tobacco mosaic virus (TMV_Cys_)^[^ ^38^ ^]^ was obtained from plants as described.^[^ ^38^, ^39^ ^]^ To bind streptavidin‐conjugated horseradish peroxidase (SA‐HRP) to TMV_Cys_ via biotin, the procedure of Koch et al. was adopted.^[^ ^39^ ^]^ 1 mL of a TMV_Cys_ suspension with a concentration of 10 mg mL^−1^ in coupling buffer (25 mm MOPS, 5 mm EDTA, 5 mm TCEP, pH 7 with 0.01% thiomersal) was supplemented with 6.83 µL of a 250 mm solution of maleimide‐PEG_11_‐biotin linker (Bio) dissolved in DMF. TMV_Cys_ and linker were coupled at 26 °C for 3 h under 800 rpm orbital agitation to yield TMV_Cys_‐Bio. The unbound linker was removed, and buffer was exchanged to 10 mm SPP by five subsequent cycles of ultrafiltration and retentate resuspension in 10 mm SPP (Amicon Ultra‐0.5 mL, 10 kDa MWCO, 13 000 g in 5430 R centrifuge, Eppendorf, Hamburg, Germany).

In order to verify the coupling efficiency of TMV_Cys_ to the linker, a 15% Mini‐SDS‐PAGE^[^ ^74^ ^]^ was conducted (Hoefer Vertical Mini Unit, 25 mA per gel). Samples were mixed with loading buffer^[^ ^74^ ^]^ and heated for 5 min at 90 °C. A sample volume corresponding to 1 µg of uncoupled TMV_Cys_ was loaded per lane, non‐coupled TMV_Cys_ served as positive control (P) and 10 mm SPP buffer as negative control (N). PageRuler Prestained Protein Marker (M1) and GE low molecular weight protein marker (M2) were used as markers. The gel was stained with colloidal Coomassie Brilliant Blue G250 (Serva Electrophoresis, Heidelberg, Germany) and the coupling efficiency was determined by comparing the intensity of the bands corresponding to the apparent molecular weights of coupled and uncoupled coat proteins of TMV_Cys_ with GelAnalyzer 19.1.^[^ ^75^ ^]^

### Coupling of TMV_Cys_ to Enzymes

Two different enzymes, SA‐HRP and penicillinase (Pen), were bound to the virus particles. For coupling SA‐HRP, the protocol specified by Koch et al. was followed.^[^ ^39^ ^]^ 353 µL of a 14 mg mL^−1^ TMV_Cys_‐Bio suspension were mixed with 326 µL of SA‐HRP solution (1 mg mL^−1^) with 321 µL SPP buffer overnight at 4 °C and 800 rpm (TMV‐HRP conjugate). The unbound enzyme was removed by ultracentrifugation (Optima L90K, 45Ti rotor, Beckmann Coulter GmbH, Krefeld, Germany, with Microfuge adapter, Beranek, Nußloch, Germany) at 35 000 rpm corresponding to 142 000 g and 4 °C for 1.5 h. As a control to evaluate the unspecific binding of the enzyme to TMV_Cys_, the SA‐HRP was also mixed with TMV_Cys_ following the aforementioned procedure but without the coupling step to the linker (TMV and SA‐HRP).

To bind the enzyme Pen covalently to TMV_Cys_, Pen was first solubilized and washed three times via ultrafiltration in 10 mm SPP buffer (Amicon Ultra‐0.5 mL, 10 kDa MWCO, 13 000 g). Afterward, it was mixed with a tenfold molar excess of a 12.5 mm maleimide‐PEG_12_‐NHS linker solution in DMSO for 30 min at RT. After removing the unbound linker via centrifugal filter units at 13 000 g, TMV_Cys_ was added, and the suspension was kept at 4 °C for 3 days (TMV‐Pen conjugate). Finally, ultracentrifugation was used as above, to separate Pen‐loaded TMV species from free Pen.

In order to investigate the coupling of the TMV_Cys_‐Bio to the enzyme SA‐HRP, TEM images were recorded. For this purpose, TMV_Cys_ and TMV‐HRP solutions with a concentration of 0.05 mg mL^−1^ were prepared in PBS and adsorbed on Formvar‐coated and carbon‐sputtered 400 mesh copper grids. They were washed three times with H_2_O and negatively stained with 2% uranyl acetate for 1 min. TEM used a ZEISS EM 10A operated at 60 kV and a 1‐megapixel camera (TRS Slowscan, Albert Tröndle Restlichtverstärkersysteme, Moorenweis, Germany).

### Coated PEG‐Based Hydrogels as Biosensors

TMV‐HRP and TMV‐Pen conjugates were incorporated in the GM solution with a concentration of 0.02 wt%. The solution was pipetted on isothiouronium‐functionalized PEGDA hydrogels, which had been swollen before (overnight) in deionized water. Two spots with a volume of 1 µL were placed on the hydrogel and cross‐linked as described before. The substrate solution for the enzyme was added either directly to the samples (no washing step) or after keeping them at 4 °C overnight and washing them for 1 week in PBS at 4 °C by changing the PBS twice a day. ABTS solution was used for the enzyme SA‐HRP and Pen 95 µm nitrocefin in 0.1 m phosphate buffer (stock solution 19 mm in DMSO) was used as a specific substrate. After removing residual water from the hydrogels, 3 mL substrate solution was added. For documentation, a picture of the hydrogels was taken directly afterward and after 20 min. After a total time of 25 min, the substrate solution was removed and the ABTS solution was used for absorption measurements at 405 nm, the unused ABTS solution served as a reference. The same experiment was performed for TMV and SA‐HRP with 0.02 wt% and SA‐HRP alone with 0.00006 wt%. Self‐implemented Python scripts were used to visualize the intensities of the green channel of the spots after adding ABTS. For this purpose, the photos of the spots were smoothed with a Gaussian filter, and the background was subtracted with a black top hat filter. The signal intensity was then extracted as the profile along the line of maximum intensity.

### Statistical Analysis

OriginPro 2019b (OriginLab) was used to perform a two‐way analysis of variance (ANOVA) with the Bonferroni post‐hoc test. Mean values were considered significantly different for *p*‐values < 0.05. All experiments were performed three times with independent samples and GM solution preparations.

## Conflict of Interest

The authors declare no conflict of interest.

## Author Contributions

Conceptualization: J.G. and A.S.; Methodology: J.G., A.S., and T.W.; Investigation: J.G., T.W., C.O.J., and D.U.; Visualization: J.G. and A.S.; Writing – original draft: J.G. and T.W.; Writing – review & editing: C.O.J., D.U., C.W., G.E.M.T., and A.S.; Supervision: A.S. and G.E.M.T.

## Supporting information

Supporting Information

## Data Availability

The data that support the findings of this study are available from the corresponding author upon reasonable request.
