# Supplementary material for: Soft Sub‐Structured Multi‐Material Biosensor Hydrogels with Enzymes Retained by Plant Viral Scaffolds
Source: Macromol Biosci. 2023 Nov 15;24(3):2300311. doi: 10.1002/mabi.202300311 (PMC13420911; doi:10.1002/mabi.202300311)
Supplement: Supplementary file 1 — Supporting Information [file MABI-24-2300311-s001.pdf]

Macromolecular Bioscience

Supporting Information

for *Macromol. Biosci.*, DOI 10.1002/mabi.202300311

Soft Sub-Structured Multi-Material Biosensor Hydrogels with Enzymes Retained by Plant Viral Scaffolds

Jana Grübel, Tim Wendlandt, Daniela Urban, Corinna O. Jauch, Christina Wege, Günter E. M. Tovar and Alexander Southan**

Supporting Information for

Soft sub-structured multi-material biosensor hydrogels with enzymes retained by plant viral scaffolds

Jana Grübel[a], Tim Wendlandt[b], Daniela Urban[a], Corinna O. Jauch[a], Christina Wege[b], Günter E. M. Tovar[a,c*], Alexander Southan[a,d*]

[a]Institute of Interfacial Process Engineering and Plasma Technology IGVP, University of Stuttgart, Nobelstr. 12, 70569 Stuttgart, Germany.

[b]Institute of Biomaterials and Biomolecular Systems, University of Stuttgart, Pfaffenwaldring 57, 70569 Stuttgart, Germany.

[c]Fraunhofer Institute for Interfacial Engineering and Biotechnology IGB, Nobelstr. 12, 70569 Stuttgart, Germany.

[d]Max Planck Institute for Intelligent Systems, Heisenbergstr. 3, 70569 Stuttgart, Germany. E-Mail: southan@is.mpg.de.

Table of Contents

1 Chemical structure of 2-(11-(acryloyloxy)undecyl)isothiouronium bromide (AUITB) 2

2 ^{1}H NMR spectrum of gelatin methacryloyl (GM10) 2

3 Viscosity and surface tension of the gelatin methacryloyl solution 2

4 Contact angle and cross-linking of gelatin methacryloyl solution on control samples 3

5 Cross-linking and multi-material combination chemistry 4

6 Coupling of cysteine-modified tobacco mosaic viruses (TMV_{Cys}) to enzymes 4

7 Enzyme activity in gelatin methacryloyl spots 5

8 Penicillinase (Pen) long-term shelf-life and reusability: activity of pre-used Pen in gelatin methacryloyl (GM) spots after 22 months of dry storage 9

1 Chemical structure of 2-(11-(acryloyloxy)undecyl)isothiouronium bromide (AUITB)

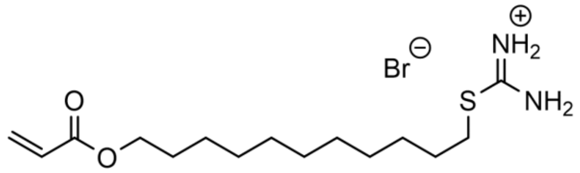

Figure S 1. Structural formula of the monomer AUITB. The monomer was copolymerized with poly(ethylene glycol) diacrylate (PEGDA) to introduce isothiouronium groups into the samples.

2 ^{1}H NMR spectrum of gelatin methacryloyl (GM10)

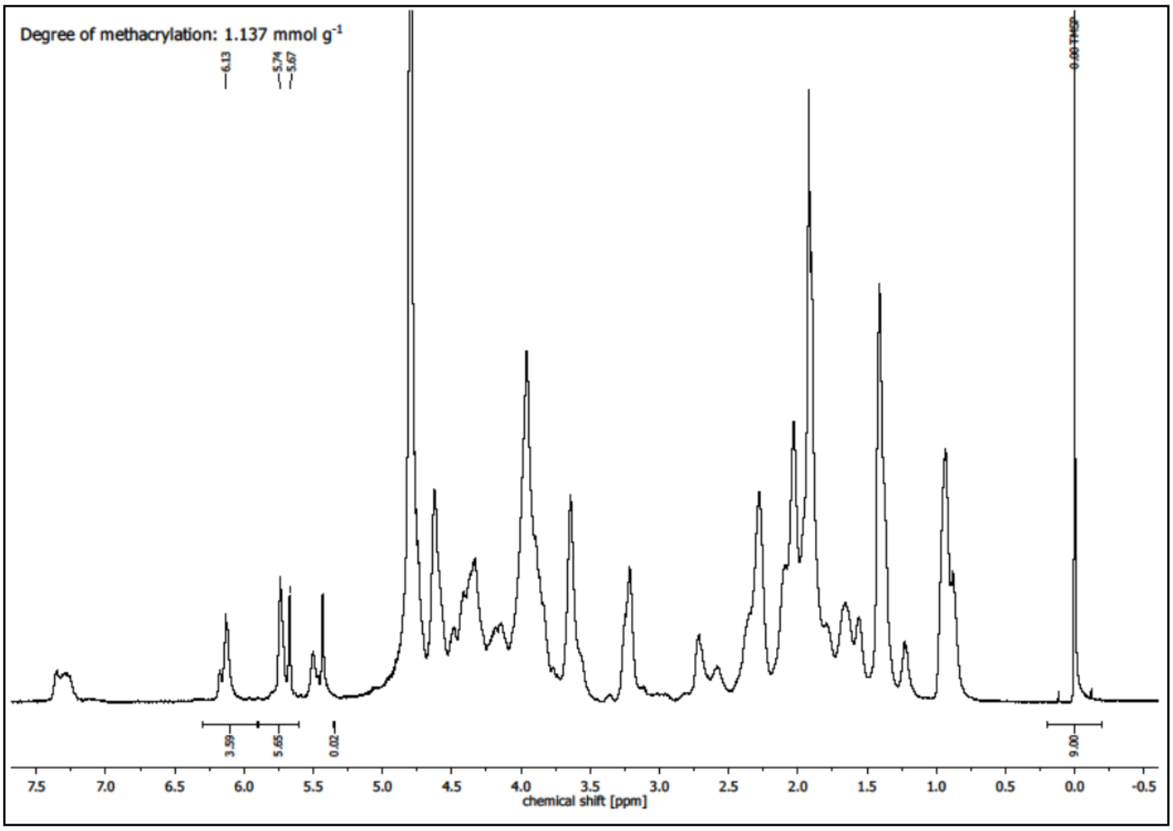

Figure S 2. ^{1}H NMR spectrum of GM10.

3 Viscosity and surface tension of the gelatin methacryloyl solution

The viscosity and the surface tension of the GM solution were examined since they are important parameters for the inkjet printing process. The viscosity of the GM solution was almost constant at shear rates between 10 s^{-1} and 1000 s^{-1} with an average viscosity of 3.20 mPa s ± 0.83 mPa s. The surface tension of the first stable bubble at 15 ms was 58.03 mN m^{-1} ± 0.38 mN m^{-1} (Figure S 3).

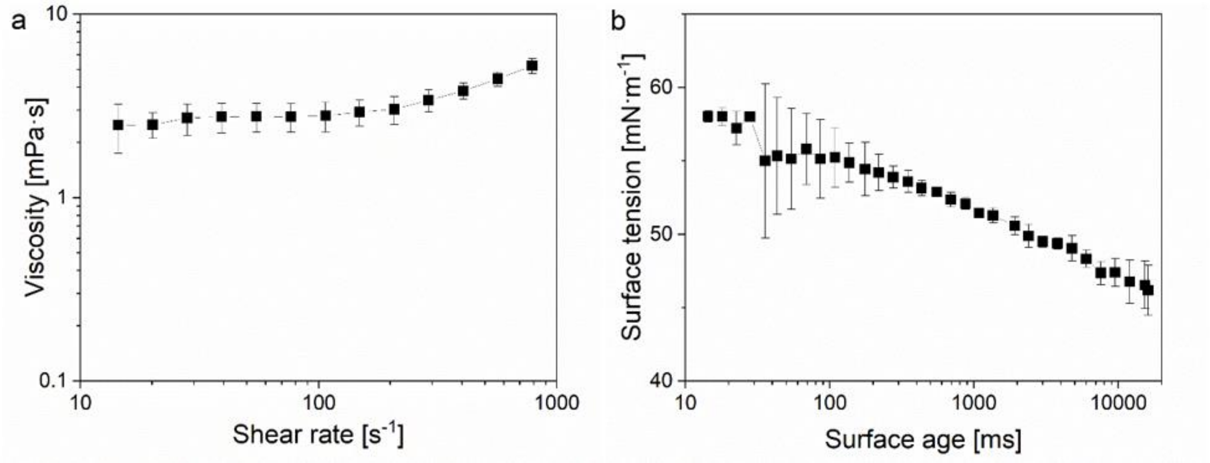

Figure S 3. Characterization of the GM solution. The solution consisted of 7.5 wt% GM with 0.7 wt% lithium phenyl-2,4,6-trimethylbenzoylphosphinate (LAP) relative to the biopolymer mass in phosphate buffered saline (PBS) pH 7.4 (n = 3). a) Viscosity of the solution against the shear rate. b) Surface tension of the solution as a function of the surface age of the bubbles.

4 Contact angle and cross-linking of gelatin methacryloyl solution on control samples

Non-functionalized PEG-based hydrogels, which were treated with sodium metabisulfite served as controls. The contact angle of the GM solution on those hydrogels was 24.1° ± 2.6° (n = 3). The surface area *A* of the cross-linked GM spots on those hydrogels is shown in Table S 1.

Table S 1. Determined surface areas *A* of the GM spots, which were cross-linked under argon atmosphere, on day 0, 7, 14 and 21 on treated, non-functionalized PEG-based hydrogels (n = 3).

Day	Surface area *A* [mm^2]
0	0.725 ± 0.315
7	0.757 ± 0.304
14	0.714 ± 0.290
21	0.674 ± 0.328

5 Cross-linking and multi-material combination chemistry

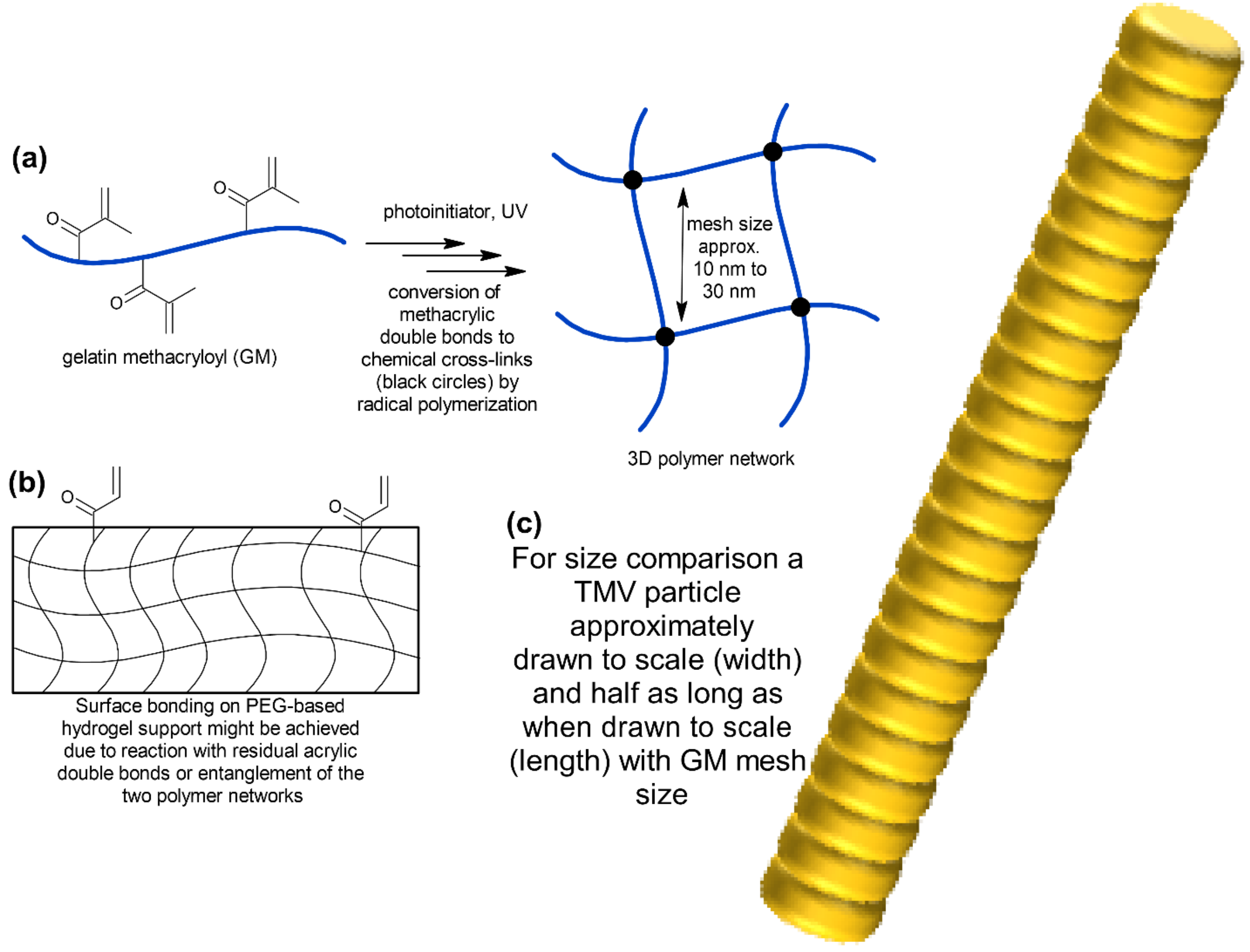

Scheme S 1. (a) Schematic representation of cross-linking chemistry during GM hydrogel curing. During curing, the methacrylic double bonds are converted to chemical cross-links so that a three-dimensional polymer network is formed. (b) Surface anchoring of GM hydrogel spots on the PEG-based hydrogel support may be achieved by reaction with residual acrylic groups or entanglement. However, the exact mechanism is unclear. (c) A TMV particle approximately drawn to scale (width) and half as long as when drawn to scale (length) with the GM mesh size. It becomes evident that the TMV particle cannot escape a fully cross-linked three-dimensional GM network by diffusion.

6 Coupling of cysteine-modified tobacco mosaic viruses (TMV_{Cys}) to enzymes

The coupling of the maleimide-PEG_{11}-biotin (Bio) linker to TMV_{Cys} was verified by SDS-PAGE and a shift of the band of the coat protein from TMV_{Cys} (17.6 kDa) was observed due to the conjugation of the linker (922 Da, Figure S 4, lower right corner). A coupling efficiency of approx. 93 % was determined. After bioaffinity coupling of the streptavidin-coupled horseradish peroxidase (SA-HRP) to the TMV_{Cys}-Bio particles, transmission electron microscopy (TEM) images were comparable with the ones before coupling, suggesting that the structure of the virus particles remained intact.

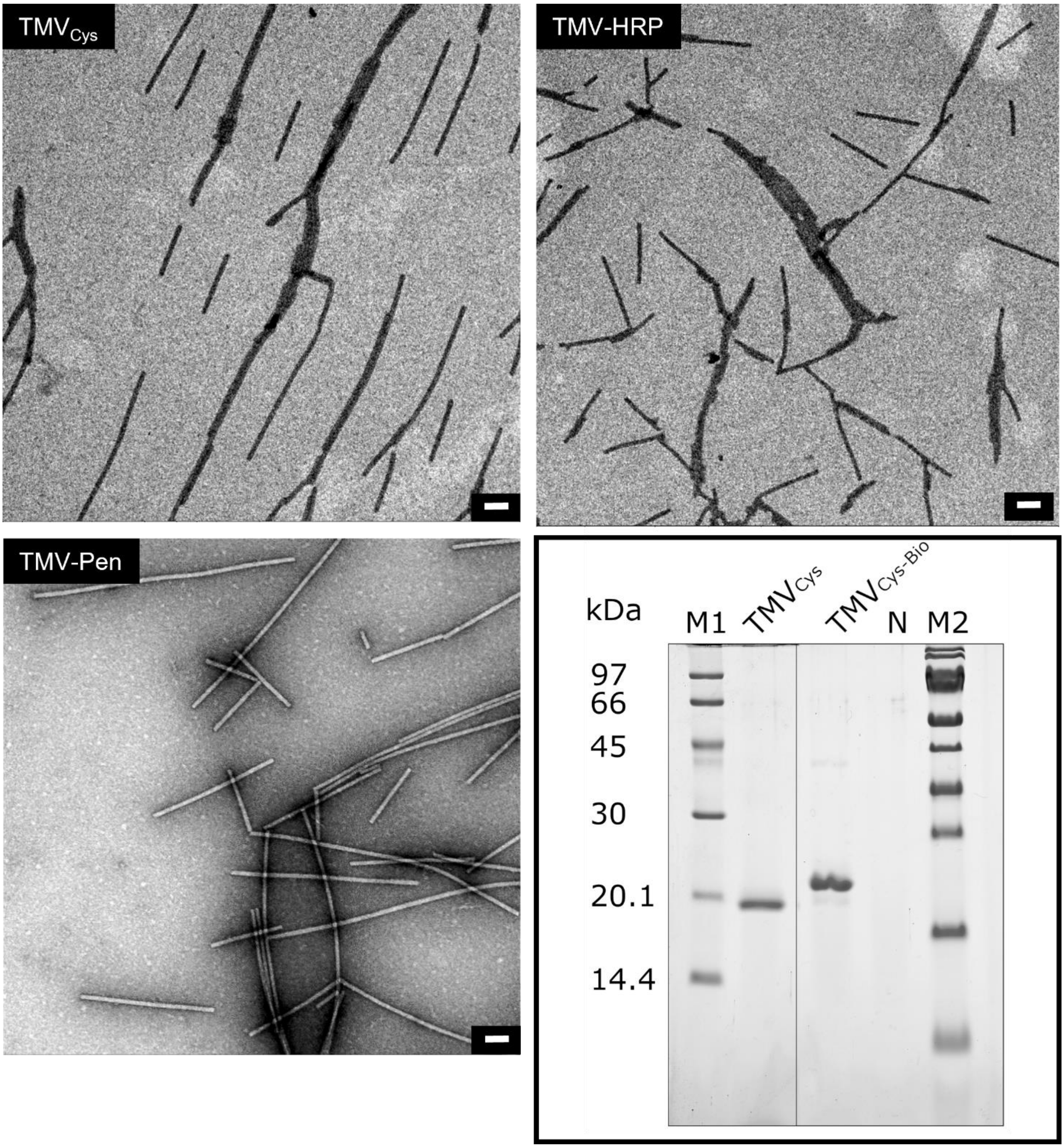

Figure S 4. Coupling of the coat protein (CP) subunits of TMV_{Cys} to enzymes. TEM images after UAc negative staining of TMV_{Cys}, TMV_{Cys} coupled to penicillinase (TMV-Pen) and TMV_{Cys} coupled to horseradish peroxidase (TMV-HRP), scale bars 100 nm. Lower right corner: 15 % SDS-PAGE to determine the coupling efficiency of TMV_{Cys} CPs to the linker (Bio). Coupling efficiencies of 93 % (CPs with biotin-terminated linkers) were obtained (TMV_{Cys}-Bio). M1 / M2: molecular mass markers, positive control (TMV_{Cys}), N: negative control: sodium-potassium-phosphate (SPP) buffer.

7 Enzyme activity in gelatin methacryloyl spots

In Figure S 5, photos of the hydrogel samples and the respective color intensity directly after adding the substrate solution (0 min) are depicted.

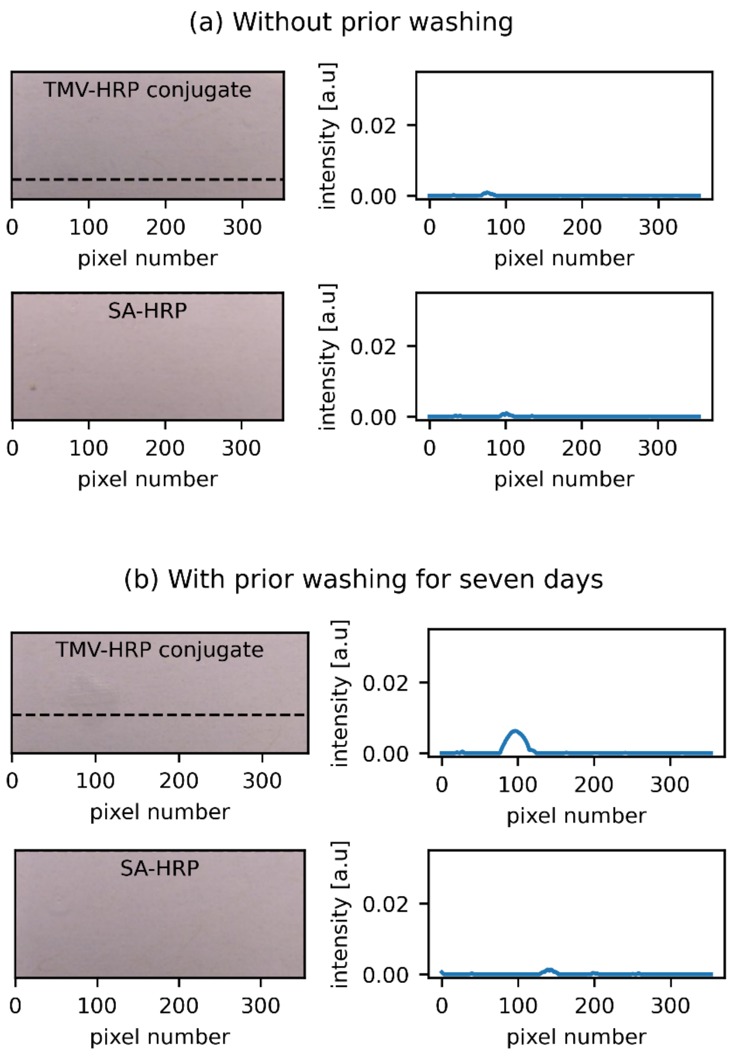

Figure S 5. Analysis of the enzyme activity in GM spots anchored on a hydrogel substrate directly after adding substrate solution (0 min). Either tobacco mosaic virus – horseradish peroxidase (TMV-HRP) conjugate or SA-HRP was integrated in the GM spot. Photos of hydrogels were taken directly after adding 2,2‘-azino-bis(3-ethylbenzothiazoline-6-sulfonic acid) (ABTS) substrate solution (0 min). Right next to the photos, the respective color intensity along the dashed line in the photos is plotted. (a) ABTS was added directly after sample preparation, *i.e.* without any washing steps. (b) After extended washing of the samples, ABTS was added. For TMV-HRP conjugate an intensity was already detected in the 0 min photo for one spot. The enzyme reaction already started probably due to time delay at capturing the image.

As a control to verify the specific binding of the linker-exposed biotin to SA-HRP, TMV_{Cys} was mixed with SA-HRP without prior addition of a biotin-linker (TMV and SA-HRP). After purification, this solution was also integrated in the GM spots to evaluate remaining enzyme activity (Figure S 6 and Figure S 7).

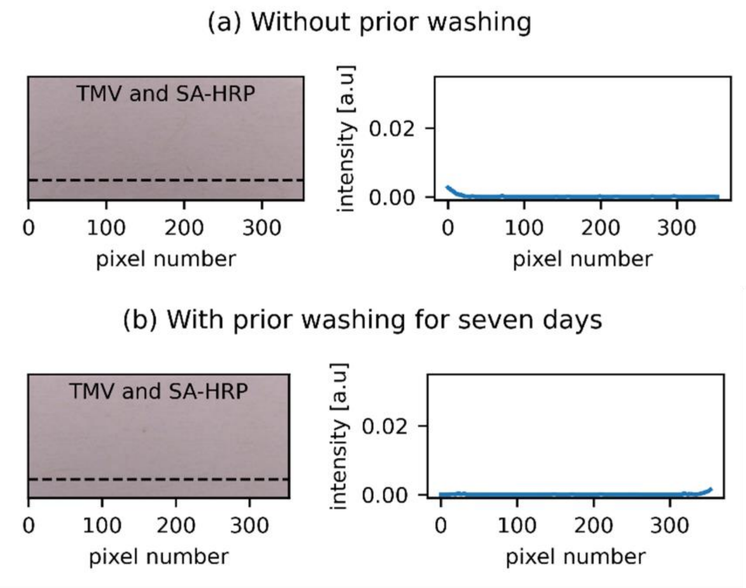

Figure S 6. Analysis of the enzyme activity in control GM spots anchored on a hydrogel substrate directly after adding ABTS substrate solution (0 min). Right next to the photos, the respective color intensity along the dashed line in the photos is plotted. (a) ABTS was added directly after sample preparation, *i.e*. without any washing steps. (b) After extended washing of the samples, ABTS was added. As expected, no intensity was detected.

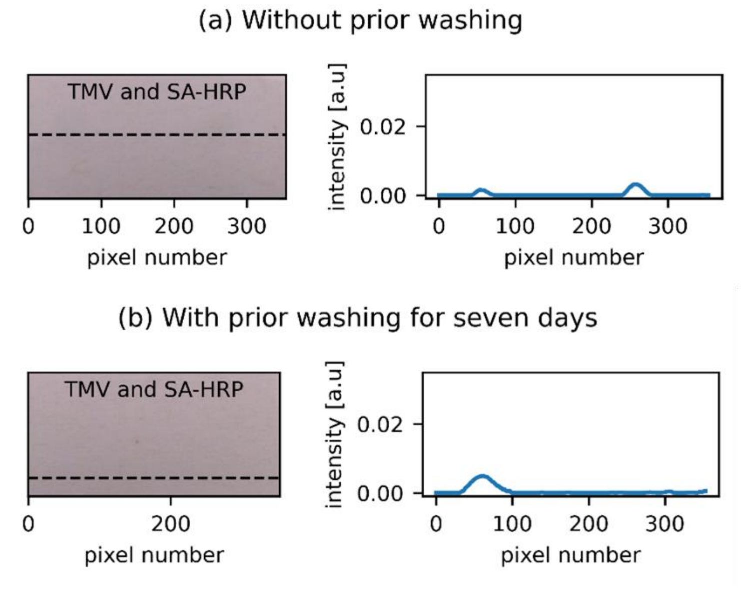

Figure S 7. Analysis of the enzyme activity in control GM spots anchored on a hydrogel substrate after offering ABTS substrate solution for 20 min. Right next to the photos, the respective color intensity along the dashed line in the photos is plotted. A slight intensity was detected indicating an unspecific binding of some enzyme molecules to the TMV particles. The color reaction was not clearly seen with the bare eye. (a) ABTS was added directly after sample preparation, *i.e*. without any washing steps. (b) After extended washing of the samples, ABTS was added.

In Table S 2 the absorption values of the ABTS solution after 25 min contact to the samples are displayed compared to the absorption values of fresh ABTS solution.

Table S 2. Absorption values of ABTS solution after 25 min. Hydrogels with GM spots incorporating TMV-HRP conjugate, TMV and SA-HRP or SA-HRP, either non-washed or washed, were kept in the substrate solution for 25 min. The corresponding absorption values of non-used ABTS solution are displayed. Values are listed as mean ± SD (n = 3).

Sample	Non-washed	washed
TMV-HRP	0.077 ± 0.004	0.063 ± 0.004
ABTS	0.059 ± 0.000	0.061 ± 0.004
TMV and SA-HRP	0.062 ± 0.001	0.065 ± 0.004
ABTS	0.059 ± 0.000	0.065 ± 0.005
SA-HRP	0.088 ± 0.003	0.059 ± 0.001
ABTS	0.059 ± 0.000	0.059 ± 0.000

For the control GM spots incorporating TMV and unbound SA-HRP, the ratio of absorption values of the ABTS solution after reaction for 25 min to fresh ABTS solution was 1.055 ± 0.020 for non-washed samples and 1.002 ± 0.008 for washed samples. This suggests that the absorption values for used and non-used ABTS solution were similar showing no distinct color reaction.

8 Penicillinase (Pen) long-term shelf-life and reusability: activity of pre-used Pen in gelatin methacryloyl (GM) spots after 22 months of dry storage

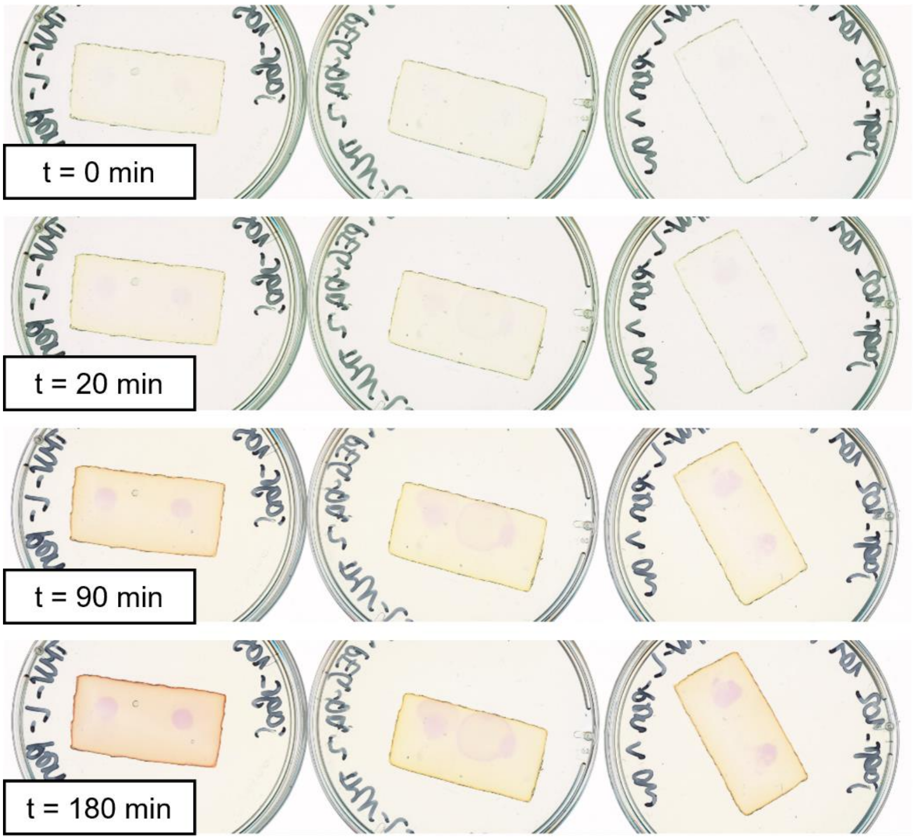

Figure S 8. Enzyme activity of hydrogel sensor with penicillinase (Pen) after 22 months of dry storage, following its preparation and initial application. Remaining enzyme activity of TMV-Pen conjugates was tested after drying, cold storage and rehydration of the hydrogel samples. After thorough washing of the re-swollen gels, nitrocefin substrate solution was added and pictures were taken at specific time points indicated at the left. Enzyme activity resulted in a red color reaction in the GM spots.
